# Diverging Mineral Chemistry of Iron and Nickel Throughout Earth’s Changing Redox Conditions Reveals Foundation for Their Evolution as Protein Cofactors

**DOI:** 10.3390/life16050747

**Published:** 2026-05-01

**Authors:** Benjamin I. Jelen, Yarissa Peralta, Shaunna M. Morrison, Beth Christensen, Eli K. Moore

**Affiliations:** 1 School of Earth and Environment, Rowan University, Glassboro, NJ 08028, USA; christensenb@rowan.edu; 2 College of Science and Mathematics, Rowan University, Glassboro, NJ 08028, USA; 3 Department of Earth and Planetary Sciences, Rutgers University, New Brunswick, NJ 08854, USA; sm3108@eps.rutgers.edu; 4 U.S. Geological Survey, Geology, Energy & Minerals Science Center, Reston, VA 20192, USA

**Keywords:** iron, nickel, minerals, proteins, early metabolism, geobio-coevolution

## Abstract

Iron (Fe) and nickel (Ni) were both foundational to early metabolism, yet their biological trajectories diverged as Earth’s surface redox state changed. Here, we integrate mineral chemistry network analysis, protein metal-site coordination-sphere analysis, and curated redox comparisons to test how geochemistry and metalloprotein architecture co-evolved. Mineral network analyses show broader electronegativity variation and network diversity for Fe-bearing minerals through time relative to Ni-bearing minerals. In structural analyses of protein metal centers in a combined Fe/Ni protein structure set, it is shown that Fe- and Ni-associated environments differ in amino-acid composition, hydropathy structure, and cysteine representation. The greater chemical diversity and electronegativity variation in Fe minerals mirror the higher redox and structural versatility of Fe-binding proteins. The presence of Fe in a broader range of mineral and protein environments demonstrates the chemical adaptability of the metal, from the anoxic Archean to oxidative Earth surface conditions following the Great Oxidation Event. Iron, with its broad redox potential range in Fe-oxidoreductases, has a central role in both anaerobic and aerobic metabolisms. Nickel, by contrast, is less widespread in biology. Today, Ni is predominantly employed in deeply branching anaerobic pathways and by proteins with narrower redox potential ranges. Our results show that evolutionary processes, constrained by metal chemistry, habitually utilize Fe as a redox generalist while retaining Ni in specialized roles. The divergent paths of Ni and Fe, from rocks to proteins, demonstrate the intimate relationship between planetary geochemistry and metabolic origins on Earth and suggest that Fe/Ni geochemistry may inform habitability assessments in extraterrestrial environments when interpreted within specific planetary environmental contexts.

## 1. Introduction

The transition metals iron (Fe) and nickel (Ni) are both crucial biological cofactors, utilized by the metabolic pathways of early life and those of the present day [1,2,3]. Sulfate reduction, sulfur reduction, anoxygenic photosynthesis (all require Fe), and methanogenesis (requires Ni and Fe) are deeply branching metabolisms in the tree of life, and geochemical evidence indicates that these pathways were present on primitive Earth at least 3.4 billion years ago (Ga) [4,5,6,7,8]. However, Fe was incorporated as a cofactor in many more metabolic pathways through time, because it could access a wider range of redox potentials and substrates than Ni as Earth’s surface became increasingly oxidized [9,10,11,12]. The disparate biological trajectories of Fe and Ni are influenced by the redox mobility, planetary interior dynamics and greater crustal abundance of Fe [13,14]. However, the physicochemical properties that directed the evolution of the chemical environments of core Fe and Ni electron transfer proteins (e.g., oxidoreductases) that impact metabolic functions in the biogeochemical electron marketplace [15] are not fully understood.

In the broader context of mineral evolution, the distinct compositions of Fe-containing minerals and Ni-containing minerals provide a record of the geochemistry and redox interactions of these two elements with their environments through time [16,17]. The proteins involved in ancient metabolisms (e.g., methanogenesis, sulfate reduction), like methyl-coenzyme M reductase and NiFe hydrogenases, that require Fe and/or Ni, typically occur in reducing environments and have negative redox midpoint potentials [vs., standard hydrogen electrode (SHE); Refs. [18,19,20]. Metabolic pathways requiring Ni are largely based on methane, carbon monoxide, and hydrogen; these pathways likely originated under the anaerobic reducing conditions of early Earth, but are rarely present in modern multicellular organisms [15,21,22,23]. Indeed, the atmosphere of early Earth, when Ni metabolisms evolved, is thought to have been hydrogen-rich [24,25]. Nickel-iron (NiFe) hydrogenases largely occur in microorganisms that consume hydrogen, and are thought to be involved in life’s most primitive metabolic pathways, utilizing hydrogen as an electron source [26,27]. Conversely, the widespread incorporation of Fe as a cofactor in both anaerobic and aerobic metabolic pathways continued throughout Earth history, including in multicellular eukaryotes, despite the decreasing bioavailability of Fe following oxygenation of the oceans [28,29]. Here, we contrast the chemical drivers behind the incorporation of Fe and Ni cofactors into proteins by looking at their presence and configuration in minerals throughout Earth’s history.

Many oxidoreductase proteins, such as NiFe-hydrogenases or ferredoxins, contain metal cofactors, such as Fe_4_S_4_ or Fe_3_NiS_4_, that resemble mineral lattices [30] [e.g., henzhuangite, NiFeS_2_; mackinawite, (Fe,Ni)_1+x_S (x = 0–0.07); greigite Fe^2+^Fe^3+^_2_S_4_; pyrite, FeS_2_]. These minerals readily form in the environment under a wide range of conditions and concentrations of constituent elements [31]. It has been proposed that prebiotic reaction networks were supported by naturally occurring mineral or metal catalysts [32], and pyrite (FeS_2_) can act as a catalyst in the formation of multiphase primitive cells [33]. There is a direct correlation between the chemical environment an organism inhabits and the redox potential of biological metal cofactors, in which low-redox-potential enzymes typically operate in reducing environments and are found in anaerobic conditions, and higher redox potential enzymes have evolved to operate in oxidized environments and are found in aerobic settings [34,35]. The repetitive crystal lattice structure and chemical bonds between different elements within minerals can inform the physical-chemical drivers of prebiotic reaction networks as well as metal cofactor incorporation into enzymes. We investigate the evolving chemistry of Fe and Ni preserved in the mineral record as a function of surface conditions by applying mineral chemistry network analysis via the novel weighted Mineral Element Electronegativity Correlation of Variation (wMEE_CV_) metric [36] to show the distinct element interactions of the two elements. We identify direct links between the wMEE_CV_ of Fe and Ni containing minerals, the hydropathy (the hydrophobicity or hydrophilicity) of Fe and Ni protein folds, and the redox potential of Fe and Ni proteins, which contributed to the divergent paths of Fe and Ni from minerals to biological protein cofactors. We also examine the implications for potential mechanisms of life on other planetary bodies.

## 2. Materials and Methods

### 2.1. Mineral Chemistry Network Analysis and Electronegativity Variation

**Figure 1:**
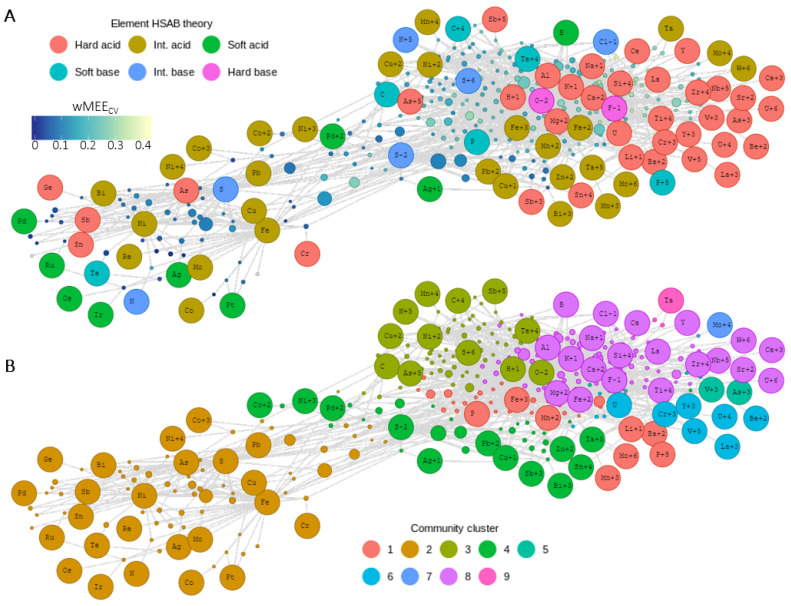
Electronegativity and hard-soft acid-base chemistry are embedded in the combined Fe and Ni mineral chemistry network. (**A**) Mineral chemistry protein cofactor network of all Fe-containing and Ni-containing minerals with maximum ages >2.5 Ga. Element nodes are separated by redox state. Mineral nodes are colored by their weighted Mineral Element Electronegativity Coefficient of Variation (wMEE_CV_) values, and elements are colored by Hard Soft Acid Base (HSAB) classification. Minerals are sized by the number of known localities. (**B**) Same network with element and mineral nodes colored by the Louvain network community [39]. Louvain network community detection groups minerals and their constituent elements into communities based on their shared network connections.

Mineral chemistry bipartite networks, which contain two types of nodes, minerals and their constituent elements (Figure 1), were constructed using the publicly available R package dragon v1.2.1 [36,37]. Data used in network analysis were obtained from the Mineral Evolution Database (MED; https://odr.io/MEDAges; accessed on 15 March 2023). The MED contains the nominal chemical formulas, known redox chemistry of mineral constituent elements, and the oldest/maximum known ages of all known mineral species. Mineral chemistry bipartite networks consist of mineral nodes and element nodes, in which mineral nodes have network connections to all of the constituent elements of that mineral (network lines are referred to as “edges”). For example, the mineral oregonite (FeNi_2_As_2_) node has network edges connected to Fe, Ni, and As. By integrating all of the chemical and redox interactions between Fe, Ni, and the other elements they form minerals with, these bipartite networks provide a detailed view of the geochemistry and redox conditions of Fe minerals and Ni minerals at the time of their formation. The default network layout and default node position use the force-directed Fruchterman-Reingold algorithm [38], which positions nodes based on the distributions of shared edges among nodes throughout the network. Mineral chemistry networks were constructed at different periods in deep time to investigate changes in Fe and Ni mineral chemistry at certain periods of the Archean. The time period of >3.5 Ga was chosen to include Fe and Ni mineral chemistry before evidence of diverse metabolisms that used Fe and Ni as protein cofactors [4,5,6,7,8], and the time period of >2.5 Ga was chosen to include Fe and Ni mineral chemistry before the GOE impacted Fe and Ni redox chemistry and incorporation as protein cofactors [12,15].

The mineral nodes within the networks are sized by the number of known localities for each mineral in order to account for the crustal distribution of different mineral species. In the >2.5 Ga mineral chemistry network, element nodes are separated by redox state to interrogate different redox associations or differences among Fe and Ni minerals. Louvain community detection analysis [39] was performed on different networks to identify shared network connections between different elements, redox states, and minerals. Community detection analysis performed on mineral chemistry bipartite networks provides greater insight into which elements form minerals with each other, identifying the relationships both analytically and visually. Louvain community detection does not distinguish between different types of network nodes, providing a mathematical foundation for mineral and element associations. Our use of the MED is intended to compare broad patterns in Fe- and Ni-bearing mineral chemistry through Earth history, rather than to reconstruct the exact mineral assemblages of any single early Earth environment. The presence of a mineral species in the MED does not imply equal abundance, bioavailability, or direct biochemical relevance in early Earth settings. Rare terrestrial phases and meteorite-associated minerals are interpreted here as part of the broader chemical space of Fe and Ni, not as evidence that they dominated the environments in which early metabolism emerged.

The weighted mineral element electronegativity coefficient of variation (wMEE_CV_) metric was calculated for each mineral species from the Pauling electronegativity values of the minerals’ constituent elements, weighted by the number of elements in the mineral [40], in order to further investigate changing chemical interactions within Fe minerals and Ni minerals through time. The Pauling electronegativity was chosen because the electronegativity values are determined using multiple different covalent bonds for a given element [41]. Each wMEE_CV_ value was derived by accounting for the total number of each element in the IMA mineral formula (Supplementary Tables S1 and S2). In the example of oregonite (FeNi_2_As_2_), the Pauling scale electronegativity values for the constituent elements are: Fe = 1.83; Ni = 1.91; As = 2.18. Oregonite contains one Fe atom, two Ni atoms, and two As atoms, totaling five atoms. Therefore, the wMEE_CV_ value is calculated from the five values 1.83, 1.91, 1.91, 2.18, and 2.18. The coefficient of variation in these five values is the standard deviation (0.166) divided by the mean (2.002), giving a value of 0.083.

The wMEE_CV_ metric provides a unique characterization of the redox interactions between all of the constituent elements in each mineral species. Challenges exist in using the standard mineral classification for investigating mineral diversity and planetary evolution [42]. Within-mineral electronegativity variation among constituent elements is a chemical feature not resolved by redox state alone, nor directly represented by broader classification frameworks. Its value is for comparison of Fe- and Ni-bearing mineral chemistry, rather than as a universal index of mineral diversity. The range of different electronegativity values among chemical elements and the diversity of different electronegativity combinations among the constituent elements of different mineral species give more nuanced information than nominal redox state [40]. Oxygen is a hard base and a highly electronegative element, and the presence of oxygen in a mineral generally makes the wMEE_CV_ value of the mineral larger because the high electronegativity value of oxygen adds more electronegativity variation in the mineral formula. Conversely, hard acids have low electronegativity values, and the presence of hard acids in a mineral also generally makes the wMEE_CV_ value of the mineral larger because the low electronegativity value of the hard acid also adds more electronegativity variation in the mineral formula. A mineral that contains both oxygen and a hard acid will generally have a high wMEE_CV_ value compared to other minerals [ex: andradite, Ca_3_Fe_2_(SiO_4_)_3_]. Minerals with intermediate electronegativity elements and no hard acids or hard bases will have lower wMEE_CV_ values (ex: oregonite, FeNi_2_As_2_, wMEE_CV_ = 0.083). Therefore, the wMEE_CV_ metric provides a detailed analysis of mineral chemical environments, beyond the redox state of the constituent elements.

In order to quantitatively compare the diversity of chemical interactions in Fe minerals vs. Ni minerals, the ranges of wMEE_CV_ values for Fe minerals and Ni minerals that include hard acids and bases, or do not include hard acids and bases, were calculated from the wMEE_CV_ maximum and minimum for each metal. The ranges were then used to calculate the Fe wMEE_CV_ hard acid/base ratio (Fe_MHR_) and Ni wMEE_CV_ hard acid/base ratio (Ni_MHR_). The wMEE_CV_ “0” values are for metallic Fe and Ni:

(1) wMEE_CV_ range of Fe minerals with hard acids and/or bases = 0.519 − 0.188 = 0.331
(2) wMEE_CV_ range of Fe minerals with no hard acids and/or bases = 0.186 − 0 = 0.186
(3) Fe_MHR_ = 0.331/0.186 = 1.780
(4) wMEE_CV_ range of Ni minerals with hard acids and/or bases = 0.404 − 0.221 = 0.183
(5) wMEE_CV_ range of Ni minerals with no hard acids and/or bases = 0.211 − 0 = 0.211
(6) Ni_MHR_ = 0.183/0.211 = 0.867

Comparing the range of different chemical interactions and electronegativity associations with respect to redox will provide a greater understanding of how Fe and Ni were incorporated into different protein binding site environments with different functions. The Fe_MHR_ and Ni_MHR_ values illustrate the difference in hard acid/base diversity of Fe and Ni.

### 2.2. Protein Fold Coordination Sphere Analysis and Comparison

The Coordination Sphere Analysis and Comparison (CSAC) workflow allows the user to analyze large sets of 3D protein files for their metal-binding coordination sphere characteristics and to perform large-scale statistical comparison of the datasets. The CSAC workflow is written in Python (Version 3.10), with all code and instructions saved in the GitHub repository at https://github.com/benijelen/CSAC (accessed on 12 March 2026). CSAC uses the Python package Bio.PDB, a parser for 3D protein files, as well as the numpy [43] and pandas [44] packages for data manipulation. Briefly, CSAC calculates the nearest atoms and then residues to each ligand, at a 5 Angstrom cutoff. Residues are given hydropathy values based on the Kyte Doolittle scale [45], and hydropathy averages and standard deviations are calculated. Frequency vectors are created, each describing a metal center in terms of its normalized amino-acid makeup (percentage of each amino acid’s contribution to the nearby residues set). These frequency vectors are used for PCA.

Protein Data Bank (PDB) structures were curated into a combined Fe/Ni CSAC input set using protein- and family-level metadata tracking. The input list to CSAC was built from a search for the following Ni-containing enzyme names, as well as Enzyme Commission (EC) numbers in the Protein Data Bank (PDB [46]): ACS (Acetyl CoA synthetase), 6.2.1.1, CODH (Carbon monoxide dehydrogenase), 1.2.7.4, LarA (lactate racemase), MCR (Methyl Coenzyme M reductase) 2.8.4.1, NiSOD (Nickel superoxide dismutase) 1.15.1.1, and NiFe-hydrogenases from a variety of organisms, 1.12.2.1; 1.12.99.6. For global level comparison, a diverse Fe-Enzyme dataset was curated and added. The finalized combined local input produced analyzable Fe and/or Ni outputs for 181 unique PDB structures. This set includes Fe-only, Ni-only, and Fe+Ni structures, enabling both within-family and global comparisons. The input set is stored in the CSAC GitHub repository (https://github.com/benijelen/CSAC; accessed on 12 March 2026).

CSAC outputs were used to compute local-environment descriptors at the PDB level, including average hydropathy, hydropathy standard deviation, and amino-acid frequency-vector terms (including cysteine fraction). The CSAC output databases and frequency vectors can be found in the CSAC repository. Analyses and inference were conducted at the PDB-level rather than metal-center-level to avoid bias toward proteins with many metal centers. For the cysteine fraction analyses, Fe vs. Ni comparisons were performed at two levels: “shared-family level”, restricted to enzyme families containing both metals (ACS, CODH, NiFe hydrogenases), then at the global level, using the full curated Fe/Ni dataset without restricting to shared families.

## 3. Results and Discussion

### 3.1. Distinct Mineral Chemistry of Fe and Ni

The ultimate dominance of Fe over Ni in Earth systems stems from its very different chemical interactions with other elements through time, interactions which have been recorded in the mineral record. Electronegativity is the tendency of an atom to attract a shared pair of electrons to itself [41] and the central determining factor in electron transfer processes between electron donors and acceptors [47]. Mineral redox chemistry is closely linked to the electronegativity of the mineral’s constituent elements, largely due to the fact that highly electronegative elements (i.e., oxygen, fluorine) are strong oxidants that influence the redox state of other elements in a given mineral. Oxygen is a constituent element in the majority of mineral species described in the MED (https://odr.io/MEDAges; accessed on 15 March 2023; Ref. [48]). The wMEE_CV_ metric allows for the quantification of intra-mineral electronegativity variation with respect to redox [40]. Given the large number of mineral species and localities in the MED, the combination of wMEE_CV_ and hard-soft acid-base (HSAB) chemistry has been used to identify global shifts in crustal chemistry during the Proterozoic [40], predict redox state in sulfur-containing minerals for which the redox state is not known [49], and track redox transitions through time for uranium-containing minerals [50]. Prior to this study, these mineral chemistry informatics approaches have not been used to study the diverging biochemical evolution of metal cofactors.

**Figure 2:**
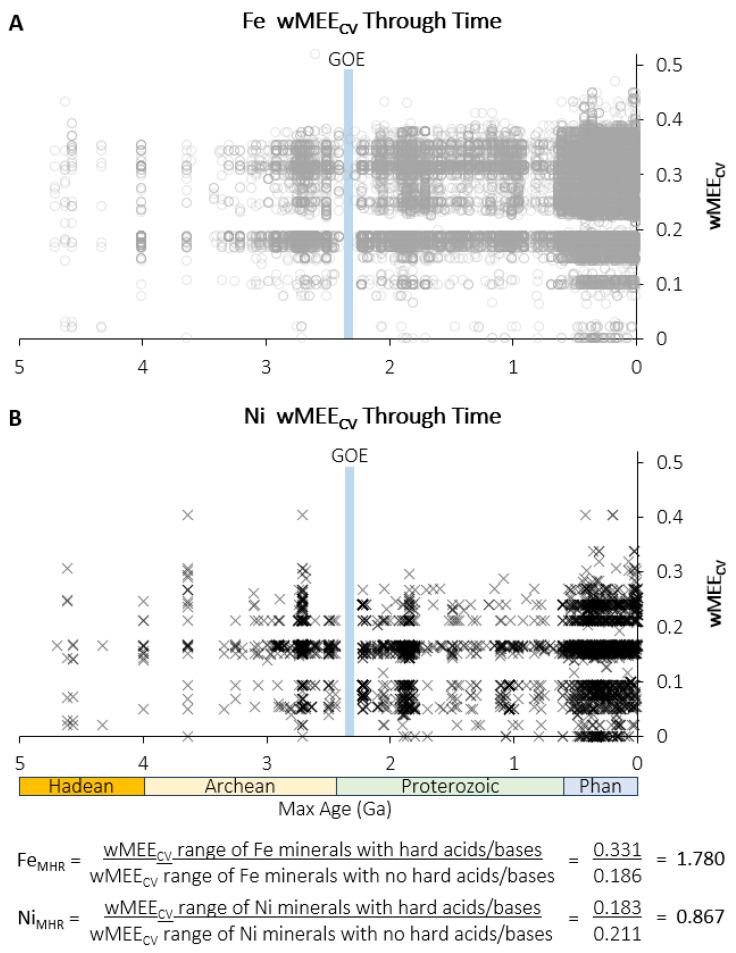
Iron and Nickel mineral chemistry electronegativity variation through time. Weighted Mineral Element Electronegativity Coefficient of Variation (wMEE_CV_) plotted by maximum known mineral age in billions of years ago (Ga) from 0 to 4.0 Ga for (**A**) Fe minerals and (**B**) Ni minerals. The range of wMEE_CV_ values becomes wider in the Proterozoic and Phanerozoic (Phan), particularly for Fe. GOE = Great Oxidation Event [8,51]. The ratio of the range of wMEE_CV_ values for minerals that contain hard acids and bases to minerals that do not contain hard acids and bases (Fe_MHR_, Ni_MHR_) is over twice as large for Fe compared to Ni.

Iron and nickel minerals preserve evidence of chemical associations at different periods of Earth history, including the Archean, during which core metabolic pathways and essential protein metal cofactors evolved [12]. But fully understanding both when and why the relative importance of Fe and Ni in different protein functions shifted remains a major challenge. We apply data analytic techniques such as network analysis and Louvain network community detection [39], which groups minerals and their constituent elements based on shared network connections, to answer this question. The combined Fe and Ni bipartite mineral redox chemistry network for minerals with maximum known ages of >2.5 Ga (e.g., Archean and Hadean; mineral ages are based on cited literature in the MED) shows that Louvain network communities align with the wMEE_CV_ differences among Fe and Ni minerals (Figure 1). Plotting wMEE_CV_ values of Fe and Ni minerals through geologic time reveals that Ni consistently shows a narrower wMEE_CV_ range than Fe (Figure 2). The majority of Fe mineral occurrences through time have wMEE_CV_ values below 0.3 (66.4%, 35,329 of 53,233), while a much larger majority of Ni mineral occurrences through time have wMEE_CV_ values below 0.3 (99.3%, 3518 of 3544). Additionally, prior to 0.6 Ga, 49.6% of Fe mineral occurrences have wMEE_CV_ values below 0.2, while a far greater proportion of Ni mineral occurrences have wMEE_CV_ values below 0.2 (74.9%) prior to 0.6 Ga. The Fe_MHR_ value is over twice as large as Ni_MHR_, representing the far greater chemical diversity of hard acids and bases in Fe minerals than Ni minerals (Figure 2). The greater chemical flexibility of Fe, exhibited by the expanded presence of hard acids and bases in Fe minerals, drives the wider wMEE_CV_ range and higher proportion of high wMEE_CV_ and hard acid/base mineral occurrences through time as compared to Ni minerals (Figure 1 and Figure 2). The intrinsic chemical differences between Fe and Ni are expressed in both mineral and protein systems, helping explain the greater diversity of Fe-bearing mineral environments, and the greater functional diversity and wider redox range of Fe-containing proteins through planetary evolution.

### 3.2. Protein Binding Site Chemical Environments and Amino Acid Distribution

**Figure 3:**
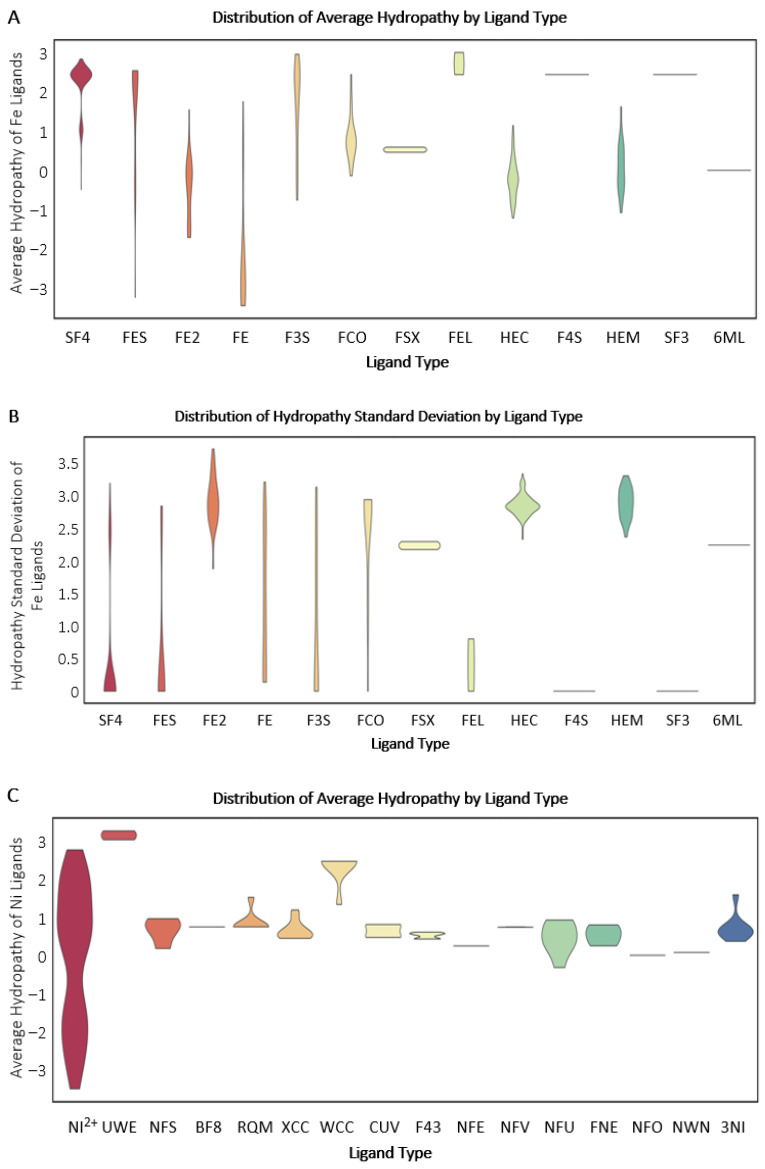

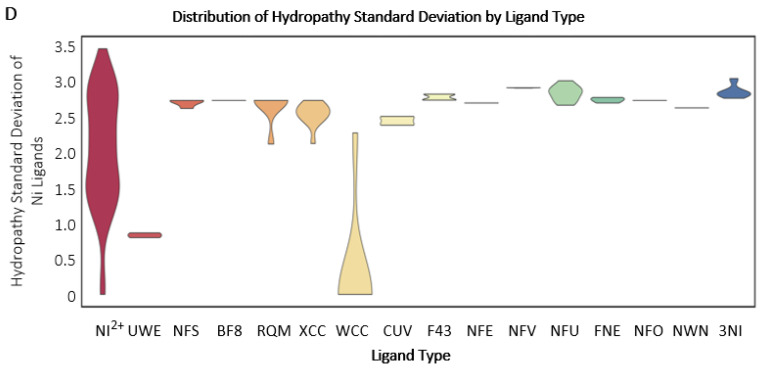
Violin plots of the distribution of protein Ni and Fe binding fold hydropathy environments. (**A**) Average hydropathy for iron (Fe) protein binding folds separated by ligand type. (**B**) Hydropathy standard deviation for Fe protein binding folds separated by ligand type. (**C**) Average hydropathy for nickel (Ni) protein binding folds separated by ligand type. (**D**) Hydropathy standard deviation for Ni protein binding folds separated by ligand type. NI^2+^: Ni^2+^; UWE: CNiO; NFS: Fe_4_-Ni-S_5_ Cluster; BF8: Fe_4_-Ni-S_5_ Cluster with Oxygen; RQM: Fe_3_-Ni-S_4_ Cluster; XCC: Fe_4_-Ni-S_4_ Cluster; WCC: FE_3_-Ni-S_4_ Cluster; CUV: Fe_4_-Ni-S_4_ Cluster, oxidized; F43: Factor 430, C_42_H_51_N_6_NiO_13_; NFE: Ni-Fe Active Center, C_2_HFeNiO_3_S_2_; NFV: Ni-Fe Oxidized Active Center, C_3_FeN_2_NiO_2_; NFU: Ni-Fe Reduced Active Center C_3_HFeN_2_NiO; FNE: C_3_FeNiO_3_S; NFO: Ni-Fe Oxidized Active Center, C_3_H_2_FeNNiO_3_; NWN: C_3_H_4_FeN_2_NiO; 3NI: NI^+3^; SF4: Fe_4_/S_4_ Iron/Sulfur Cluster; FES: Fe_2_/S_2_ (Inorganic) Cluster; FE2: Fe (II) Ion; FE: Fe (III) Ion; F3S: Fe_3_-S_4_ Cluster; FCO: Carbon Monoxide-(Dicyano) Iron; FSX: (Aqua)(Glu-O)Iron(II); FEL: Hydrated Fe; HC1: 2 Iron/2 Sulfur/5 Carbonyl/2 Water Inorganic Cluster; HEC: 2 Iron/2 Sulfur/3 Carbonyl/2 Cyanide/Water/Methyl-ether Cluster; F4S: Fe_4_-S_3_ Cluster; HEM: Protoporphyrin IX Containing Fe (Heme Group); SF3: Fe_4_-S_3_ CLUSTER; 6ML: oxygen-damaged-SF4.

Earth’s history has been directed by multiple core factors, including elemental chemistry. Minerals, biological precursors, and proteins involved in deeply branching metabolic pathways were subjected to equivalent environmental conditions that influenced their formation and function [52,53]. The hydropathy (high hydropathy = hydrophobic, low hydropathy = hydrophilic) of protein metal-binding folds can be used to compare the immediate chemical environment of metal-binding protein folds or metal-containing ligands [45]. Comparing proteins that bind Fe- versus Ni-containing ligands reveals distinct patterns that highlight the interplay through evolution between geochemistry and biology. Iron-binding folds exhibit a much wider range of average hydropathy and hydropathy standard deviation values than those binding Ni (Fe, Figure 3A,B; Ni, Figure 3C,D), suggesting greater chemical variability surrounding Fe sites compared to more constrained or uniform chemical environments around Ni sites. The ubiquity of Fe is exemplified by the far greater number of Fe-protein fold functions and structures (over 12,900 entries) than Ni (less than 2500 entries) present in the Protein Data Bank [46].

**Figure 4:**
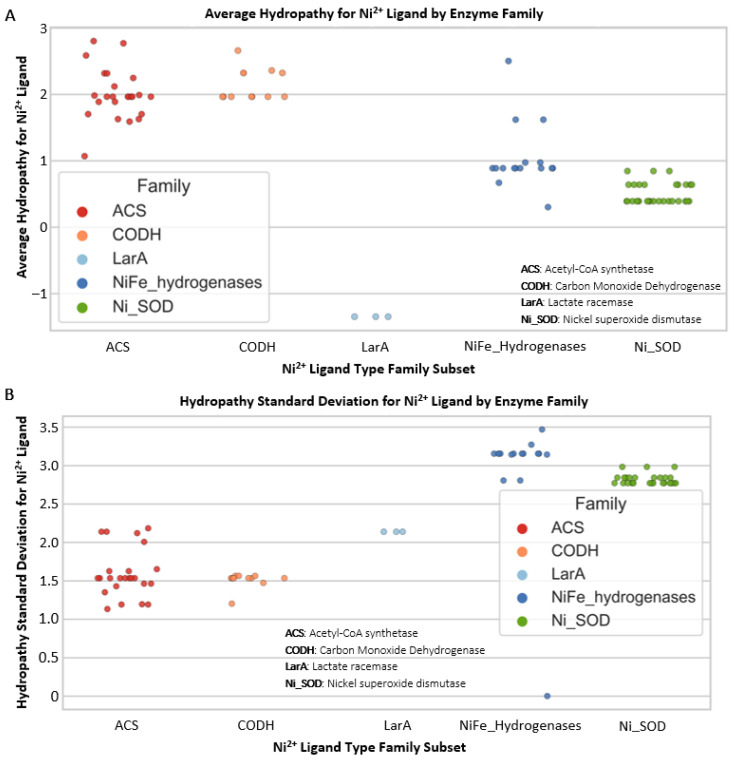
Protein Ni^2+^ binding fold hydropathy environments. Specific Ni^2+^ values from Figure 3C,D. (**A**) Distribution of average hydropathy for nickel(II) (Ni^2+^) protein binding fold ligand types from Figure 3. (**B**) Distribution of hydropathy standard deviation for nickel(II) (Ni^2+^) protein binding fold ligand types. ACS: Acetyl-CoA synthetase; CODH: Carbon Monoxide Dehydrogenase; LarA: Lactate racemase; Ni_SOD: Nickel superoxide dismutase.

As is commonly the case, there is an exception to our general finding that Ni metal-binding sites have lower hydropathy ranges and standard deviation (SD) values than Fe (Figure 3). Nickel(II) (Ni^2+^) protein folds have wide ranges of average hydropathy and hydropathy SD (Figure 3C,D). The Ni^2+^ protein fold hydropathy values are highest for Acetyl-CoA-synthetase (ACS) and carbon monoxide dehydrogenase. Looking specifically at the average hydropathy values and hydropathy standard deviation values for Ni^2+^ protein folds, the NiFe hydrogenases and superoxide dismutases have mostly intermediate values, with lactate racemase (LarA) at the lowest (Figure 4). Protein folds containing Ni^2+^ predominantly exhibit an inverse relationship between their average hydropathy values and SD distributions. A low standard deviation of hydropathy for a metal’s coordination sphere represents a homogenous chemical environment throughout the binding site and is thus more evolutionarily “rigid” in terms of chemical interactions within the sphere (fewer options for interactions means less evolvability). A higher SD, as observed for Fe coordination spheres, indicates a more heterogeneous environment at the binding site, with higher flexibility to evolve (higher heterogeneity, more evolutionary options) in response to new chemical interactions, substrates, and large-scale environmental (e.g., redox) changes. Despite the flexibility of Ni within specific protein coordination spheres, as demonstrated by its high hydropathy SD in NiFe hydrogenases, it is not as widely used in today’s biology as is Fe^2+^.

**Figure 5:**
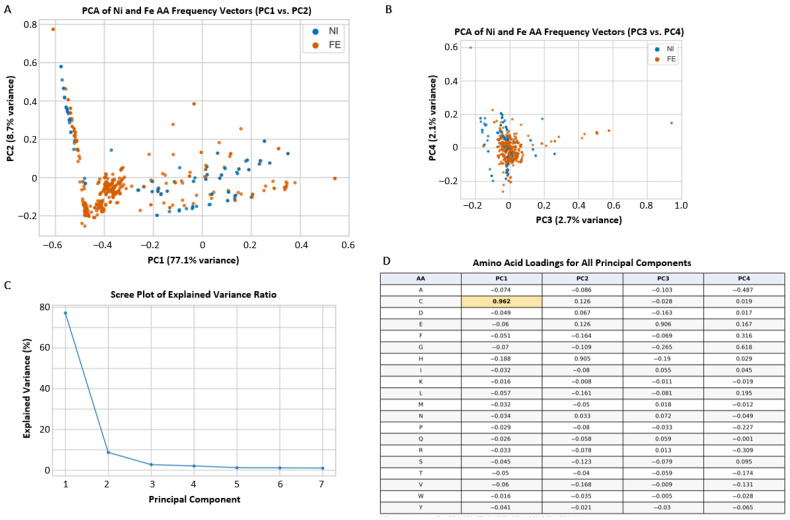
Principal component analysis (PCA) of iron (Fe) and nickel (Ni) binding protein fold amino acid (AA) frequency vectors. (**A**) Plot of principal component 1 (PC1) and PC2. (**B**) plot of PC3 and PC4. (**C**) Scree plot of explained variance ratio for each principal component. (**D**) Amino acid loadings for all principal components. The bold value for C in PC1 represents a statistically significant difference in cysteine usage between Fe and Ni folds. Amino acid abbreviations are represented by single letters.

**Figure 6:**
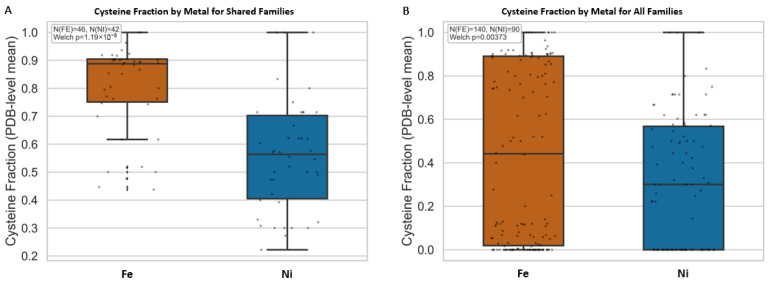
Box plot comparison of cysteine (C) loading values from the amino acid (AA) frequency vector of Fe and Ni protein binding folds for (**A**) shared Fe and Ni protein families, and (**B**) all Fe and Ni protein families. There is a statistically significant difference between the C values of Fe protein binding folds and Ni protein binding folds for both plots.

Principal component analysis (PCA) of amino acid vectors of Fe and Ni binding protein folds allows for further interrogation of the chemical environment surrounding Fe and Ni ligands (Figure 5). Differences among principal components (PC) 1 and 2 are influenced primarily by specific amino acids. Cysteine (C) is the main driver of PC1, which explains approximately 77.1% of the variance in the amino acid vector dataset. A Welch’s t-test comparing the cysteine usage (normalized percentages of amino acid character, refer to methods) showed significantly higher cysteine usage in Fe-associated folds than in Ni-associated folds in both analyses, shown in Figure 6: shared-family comparison (*p* = 1.19 × 10^−8^) and global comparison (*p* = 3.73 × 10^−3^). Cysteine’s thiol (-SH) group is one of the strongest ligands in protein coordination chemistry and is especially effective at stabilizing metals in redox-active states [54]. Indeed, the thiol groups on cysteine residues are the most common metal-binding sites in metalloenzymes [55,56]. The higher abundance of cysteine in Fe-associated sites is consistent with Fe frequently being embedded in sulfur-rich catalytic scaffolds, including in Fe-S contexts after the Great Oxidation Event (GOE [57]), whereas lower cysteine abundance in Ni-associated sites is consistent with a narrower set of recurring Ni coordination motifs across enzyme families. This does not imply that Ni lacks sulfur coordination, nor that Fe effects are universal across all families; rather, it indicates that the aggregate Fe-versus-Ni cysteine contrast emerges from how each metal is recurrently deployed in catalytic architecture over evolutionary time.

### 3.3. Iron and Nickel Chemical Associations and Redox Potentials Within Minerals and Proteins Through Time

**Figure 7:**
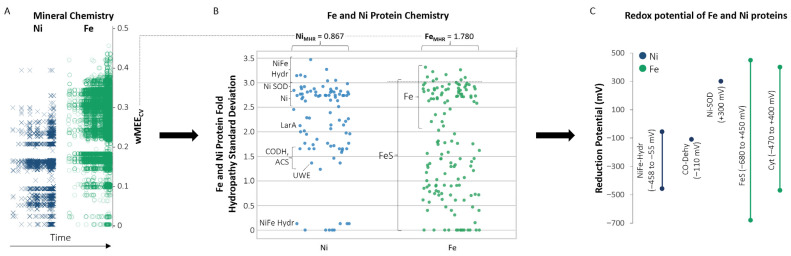
Iron (Fe) and nickel (Ni) mineral electronegativity, protein fold hydropathy, and protein redox connections. (**A**) Plots of Ni and Fe weighted Mineral Element Coefficient of Variation (wMEE_CV_) values through time (time axis progresses from past to present). (**B**) Plots of Ni and Fe protein fold hydropathy standard deviation values. (**C**) Redox potentials of Ni-and Fe-containing proteins. Fe_MHR_ = Iron wMEE_CV_ Hard acid/base Ratio; Ni_MHR_ = Nickel wMEE_CV_ Hard acid/base Ratio. NiFe Hydr: NiFe hydrogenase; Ni-SOD: Ni-superoxide dismutase; Ni: Ni^+2^; CO-Dehy = Carbon Monoxide Dehydrogenase; LarA: Lactate Racemase; UWE: CNiO; ACS: Acetyl CoA Synthetase; FeS = FeS proteins; Cyt = cytochrome proteins. Redox potentials are compiled from [11,18,19,20,65].

**Figure 8:**
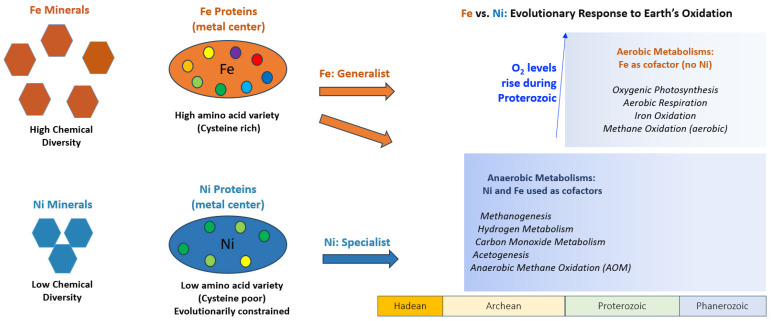
The diverging paths of iron (Fe) and nickel (Ni) from minerals to proteins. Fe-based metabolisms flourish both before and after Earth’s oxygenation. Ni-based metabolisms are almost exclusively anaerobic, reflecting their origin in the reducing Archean. Fe is used by biology as a generalist, due to its flexibility, which can be seen both in its diverse mineral chemistry and in metal-binding centers of proteins. Ni is less flexible mineralogically, chemically in proteins, and more evolutionarily constrained, as reflected by its metabolic exclusion from oxidized environments.

Environmental factors drive change in the chemistry of the geosphere and biosphere. The connection from mineral chemistry to protein metal binding folds to protein redox potential and function can be observed through the greater electronegativity variation, including the greater Fe_MHR_ value than Ni_MHR_, protein fold hydropathy variation, and redox potential range of Fe-containing oxidoreductase proteins as compared to Ni-containing oxidoreductase proteins (Figure 7). This connection can be traced through Earth’s changing redox conditions. Following seawater oxygen oscillations from 2.65 to 2.4 Ga, the GOE at approximately 2.4 Ga marked a significant increase in atmospheric oxygen, produced by cyanobacteria in the oceans [8,51,58,59]. Following the widespread anoxic conditions of the Archean (Figure 8), the rise in oceanic oxygen led to Fe and Ni precipitating as insoluble iron oxides and nickel oxides in shallow marine environments (ex., Ni(OH)_2_/NiOOH; magnetite, Fe_3_O_4_ formation following precipitation of Fe^3+^ oxyhydroxides [60,61]) and sulfides in deeper anoxic marine sediments (ex., pentlandite, (Fe,Ni)_9_S_8_; millerite, NiS; pyrite, FeS_2_), reducing both metals’ bioavailability in seawater [28,29]. Similarly, it has been proposed that the decline in Ni availability due to changes in mantle chemistry in the Archean would have adversely affected methanogens, which depend on Ni for the function of methyl-coenzyme M reductase (MCR). This “nickel famine” was also impacted by a reduction in crustal Ni sources [62,63]; it is one illustration of metals’ importance in the enzymatic production of gases, including those that profoundly affect our atmosphere and climate. Whereas the aerobic world selected against general Ni usage due to its lower concentration in oxidative environments, Fe usage was retained by biological enzymes, due in large part to Fe-scavenging mechanisms (e.g., siderophores [64]), throughout the evolutionary changes brought on by the GOE (Figure 8). Nickel redox functionality was crucial in early Earth biochemistry but was largely abandoned in newer aerobic systems, which came to favor more widely functional and abundant metals like Fe [62].

The ability of Fe to readily switch between Fe^2+^ and Fe^3+^ oxidation states under physiological conditions made it ideal for a wide range of electron transfer processes in the post-GOE world, including aerobic respiration and photosynthesis [13]. This redox functionality is partly enabled by a wide span of highly tunable and accessible redox potentials. Iron-sulfur clusters, in their various forms, span over 1 volt in redox potential (from −700 mV to +400 mV vs. SHE [11]). With this redox flexibility, Fe was favored early in electron-transport systems based on Fe-S clusters, NiFeS clusters, or Fe alone, leading to a selective advantage for Fe-containing oxidoreductases. Anomalous carbon isotope records in the Proterozoic further reflect increasing environmental oxidation state and the expansion of microbial producers and utilizers of O_2_ and SO_4_ [8,66]. Proterozoic oxygenation of Earth’s surface and ocean interior also reshaped the sulfur cycle [67], and these broad environmental changes influenced protein evolution [53], including the contrasting cofactor-binding patterns observed in Fe- and Ni-containing proteins. Even as Fe became less bioavailable in oxidized oceans [57,68], it remained the dominant metal in metalloenzymes, due in large part to its redox functionality, including in aerobic life [9,10,11,12]. At least 300 unique enzymes involved in Fe^3+^/Fe^2+^ cycling [13] have been annotated in the PDB, along with a large set of additional mechanisms and enzymes that manage oxygen sensitivity [69].

The vast majority of Ni oxidoreductases function at negative potentials and are therefore limited to anaerobic metabolic processes (Figure 7). A notable exception is Ni superoxide dismutase, which has a mostly positive redox potential range and an optimal potential of +300 mV vs. SHE [70]. Nickel superoxide dismutase, with its unique positive midpoint potential, may have evolved in response to Fe scarcity in oxidized marine environments leading up to and following the GOE [9,71]. In contrast, Fe oxidoreductases span both negative and positive redox ranges and participate in a wide variety of anaerobic and aerobic pathways, again reflecting the greater redox versatility of Fe. At alkaline pH and low concentrations of cysteine, FeCl_3_, and Na_2_S, Fe-S clusters are naturally formed and coordinated by the thiolate side chains of cysteine residues [72], representing electron carriers that can be inserted into a variety of proteins for a wide range of enzyme midpoint potentials and functions. Iron- and nickel-containing NiFe hydrogenases are especially important for understanding the evolutionary paths of these two metals because they integrate both within a single catalytic system [53,73]. The role of NiFe hydrogenases in hydrogen metabolism and primitive energy conservation helps explain their retention across the tree of life. In these enzymes, Ni remains central to catalytic hydrogen turnover under strongly reducing conditions, whereas Fe and Fe-S clusters support electron transfer across a broader structural and redox context. Different NiFe hydrogenases exhibit both high and low hydropathy standard deviation values while generally maintaining negative redox potentials, making them a distinctive group among Ni- and Fe-containing oxidoreductases (Figure 7). NiFe hydrogenases help illuminate the transition from specialized Ni-dependent anaerobic catalysis to the broader expansion of Fe-based electron-transfer biochemistry.

As Earth’s surface became oxygenated, bioavailability of multiple metal cofactors became reduced, including Fe and Ni, but the multi-functional utility of Fe and Fe-scavenging/storage mechanisms helped support the evolution of Fe-based enzymes that would become central to aerobic respiration and electron transport (Figure 8; Refs. [12,15]). For example, the heme groups in cytochromes, ubiquitous in mitochondria, bacteria, and archaea, facilitate electron transfer through redox cycling of Fe^2+^ ←→ Fe^3+^ [74]. These cytochromes play essential roles in oxidative phosphorylation, photosynthesis, and various enzymatic redox reactions, ultimately driving ATP synthesis for a large segment of life [75]. Despite being less favored from an evolutionary standpoint in aerobic metabolisms as Earth oxygenated, Ni persists in enzymes with precise catalytic demands, such as NiFe hydrogenases for hydrogen oxidation and MCR in methane biosynthesis. These enzymes illustrate the unique utility of Ni in anaerobic niches, where its stable binding and other physical-chemical properties excel in catalyzing early biochemical reactions in environments with little selective pressure from oxygen. Notably, the F430 cofactor used in MCR has no known iron equivalent, reinforcing the evolutionary retention of Ni in specific, irreplaceable roles. Analogous to the potential role of diverse Fe-chemistry as a crucial component for life beyond Earth [13], Ni complexes could serve as a fingerprint for possible anaerobic life on other planets or other potentially habitable worlds.

Various factors, such as the nuclear stability of Fe in fusion reactions and the greater abundance of Fe than Ni in the crust, suggest that Fe would be abundant in the crust of other solid planets, potentially pointing to Fe as a common cofactor in prebiotic reactions or metabolic pathways beyond Earth [13,76]. The Fe and Ni-containing phosphide mineral schreibersite (Fe_2_NiP) has been commonly found in meteorites and has been speculated to have a potential role in the origin of life [3]. Additionally, Ni and other trace metals and nutrients are suspected to exist in fluids present on Enceladus, Europa, and elsewhere [77,78]. The presence of Fe, Ni and other trace metals and metallic core-mass fraction may be an observable trait of exoplanets that helps determine their potential to support life [79,80]. Transition metal cofactors could drive electron transfer metabolic pathways on other worlds, including the use of Fe and Ni in methanogenesis or other hydrogen-requiring pathways [81,82,83]. While the roles of Fe and Ni are vastly different on an oxidized planet with multicellular aerobic life, the two elements may be equally important in anaerobic environments that make up the vast majority of known planetary bodies with the potential to support microbial life. Investigating the roles of Fe and Ni in supporting life beyond Earth must be viewed in the context of specific planetary environmental settings in order to avoid overgeneralization.

## 4. Conclusions

This is the first study linking the variation in electronegativity within minerals through deep time to the chemical environment of protein metal binding folds in order to understand geochemical drivers involved in the evolution of protein function. A common electronegativity/hydropathy/redox trend is observed between Fe and Ni in minerals and proteins. Iron-containing minerals exhibit more diverse chemical interactions and cover a wider range of wMEE_CV_ values than Ni-containing minerals. Moreover, Fe protein folds have greater amino acid variability and cover a wider range of hydropathy standard deviation values than Ni protein folds, and Fe-containing oxidoreductases cover a wider range of redox potentials than Ni-containing oxidoreductases (Figure 7). In agreement with this trend, the Ni proteins with the highest hydropathy standard deviation values are NiFe hydrogenases, which contain more Fe atoms than Ni atoms. The mineral chemistry associations of Fe and Ni correlate with differences in their respective protein reduction potentials as well. The wider range of wMEE_CV_ values for Fe mineral occurrences aligns with the wider range of midpoint redox potentials for Fe oxidoreductases, compared to their Ni counterparts [9,10,11,15,18,19,20]. The different paths in biological space taken by Fe and Ni reflect not only the geochemical constraints of metal availability and element interactions in the environment, but also the biophysical constraints of redox flexibility, coordination geometry, ease of varied natural cluster formation with amino acids, and oxygen sensitivity (Figure 8; [55,56]). Deep-time mineral informatics reveals the chemical flexibility of Fe and specialization of Ni recorded in Earth’s crust that governed their biological roles in metalloproteins. The correlation between geochemical context and protein function throughout Earth history underscores a fundamental truth: evolution does not transcend the physical-chemical properties of matter; it works within its bounds. We interpret Fe/Ni divergence as an emergent geobiological outcome of coupled metal chemistry, protein architecture, and geological (mineralogical) history. A clearer understanding of this relationship on Earth improves our ability to identify the conditions and chemical building blocks that could support life elsewhere.

## Data Availability

The mineral chemistry network analysis data in this study are available in the Mineral Evolution Database: https://odr.io/MEDAges; accessed on 15 March 2023. The protein structure data in this study are available in the Protein Data Bank: https://www.rcsb.org/; accessed on 12 March 2026.

## Supplementary Materials

The following supporting information can be downloaded at: https://www.mdpi.com/article/10.3390/life16050747/s1. Table S1: List of Fe-containing minerals in Figure 1; Table S2: List of Ni-containing minerals in Figure 1.

## Code Availability Statement

The Coordination Sphere Analysis and Comparison (CSAC) workflow code used in this study is available in the GitHub repository at https://github.com/benijelen/CSAC; accessed on 12 March 2026.

## Abbreviations

The following abbreviations are used in this manuscript:

MED	Mineral Evolution Database
wMEE_CV_	Weighted Mineral Element Electronegativity Coefficient of Variation
IMA	International Mineralogical Association
CSAC	Coordination Sphere Analysis and Comparison
