# Supplementary material for: Diverging Mineral Chemistry of Iron and Nickel Throughout Earth’s Changing Redox Conditions Reveals Foundation for Their Evolution as Protein Cofactors
Source: Life (Basel). 2026 May 1;16(5):747. doi: 10.3390/life16050747 (PMC13208631; doi:10.3390/life16050747)
Supplement: Supplementary file 1 [file life-16-00747-s001.zip › life-4235375-supplementary.pdf]

A Tale of Two Metals: The Diverging Paths of Nickel and Iron from Rocks to Proteins

Supplementary Information

Benjamin I. Jelen[1*], Yarissa Peralta[2], Shaunna Morrison[3], Beth Christensen[1], Eli K. Moore[4*]

[1]Rowan University, School of Earth and Environment, Glassboro, New Jersey, USA.

[2]Rowan University, College of Science and Mathematics, Glassboro, New Jersey, USA

[3]Rutgers University, Department of Earth and Planetary Sciences, New Brunswick, New Jersey, USA

[4]U.S. Geological Survey, Geology, Energy & Minerals Science Center, Reston, Virginia, USA

*Correspondence: jelen@rowan.edu; ekmoore@usgs.gov

Supplementary Table S1: List of Fe-containing minerals with maximum known ages >2.5 Ga that are present in the Fig. 1a and Fig. 1b networks. Weighted Mineral Element Electronegativity Coefficient of Variation (wMEE$_{CV}$). Minerals with maximum known ages (billions of years ago, Ga) greater than 4.33 Ga are from meteorites.

Fe Mineral	Chemical Formula	wMEE$_{CV}$	Maximum Known Age (Ga)
Augite	(Ca,Mg,Fe^2+^,Fe^3+^)_2_(Si,Al)_2_O_6_	0.446	4.70
Chromite	Fe^2+^Cr^3+^_2_O_4_	0.425	4.70
Cronstedtite	(Fe^2+^,Fe^3+^)_3_(Si,Fe^3+^)_2_O_5_(OH)_4_	0.307	4.70
Greenalite	(Fe^2+^,Fe^3+^)_2-3_Si_2_O_5_(OH)_4_	0.307	4.70
Hexamolybdenum	(Mo,Ru,Fe,Ir,Os)	0.076	4.70
Magnetite	Fe^2+^Fe^3+^_2_O_4_	0.393	4.70
Pentlandite	(Ni,Fe)_9_S_8_	0.196	4.70
Pyrrhotite	Fe^2+^_5_Fe^3+^_2_S^2-^_8_	0.208	4.70
Schreibersite	(Fe,Ni,Cr)_3_P	0.096	4.70
Tochilinite	6(Fe^2+^_0.9_S^2-^)Â·5[(Mg,Fe^2+^)(OH)_2_]	0.354	4.70
Troilite	Fe^2+^S^2-^	0.241	4.70
Awaruite	Ni_3_Fe	0.030	4.62
Barringerite	Fe_2_P	0.096	4.62
Cohenite	Fe_3_C	0.232	4.62
Daubreelite	Fe^2+^Cr^3+^_2_S^2-^_4_	0.242	4.62
Goethite	Fe^3+^O(OH)	0.339	4.62
Haxonite	(Fe,Ni)_23_C_6_	0.188	4.62
Hematite	Fe^3+^_2_O_3_	0.432	4.62
Isocubanite	Cu^1+^Fe^2+^Fe^3+^S^2-^_3_	0.179	4.62
Lawrencite	Fe^2+^Cl_2_	0.377	4.62
Mackinawite	(Fe,Ni)_1-1.07_S	0.196	4.62

Maghemite	(Fe^3+^_0.67_[box]_0.33_)Fe^3+^_2_O_4_	0.432	4.62
Reevesite	Ni^2+^_6_Fe^3+^_2_C^4+^O_3_(OH)_16_Â·4H_2_O	0.274	4.62
Roaldite	(Fe,Ni)_4_N	0.299	4.62
Taenite	(Fe,Ni)	0.030	4.62
Tetrataenite	FeNi	0.030	4.62
Trevorite	Ni^2+^Fe^3+^_2_O_4_	0.379	4.62
Wustite	Fe^2+^O	0.432	4.62
Andradite	Ca_3_Fe^3+^_2_(SiO_4_)_3_	0.498	4.57
Coulsonite	Fe^2+^V^3+^_2_O_4_	0.431	4.57
Hercynite	Fe^2+^Al_2_O_4_	0.436	4.57
Kirschsteinite	CaFe^2+^SiO_4_	0.498	4.57
Pigeonite	(Mg,Fe^2+^,Ca)SiO_3_	0.496	4.57
Rhonite	Ca_4_(Mg_8_Fe^3+^_2_Ti^4+^_2_)O_4_[Si_6_Al_6_O_36_]	0.434	4.57
Wairauite	CoFe	0.019	4.57
Aenigmatite	Na_4_(Fe^2+^_10_Ti^4+^_2_)O_4_[Si_12_O_36_]	0.481	4.57
Armalcolite	(Mg,Fe^2+^)Ti^4+^_2_O_5_	0.475	4.57
Fayalite	Fe^2+^_2_SiO_4_	0.381	4.57
Hedenbergite	CaFe^2+^Si_2_O_6_	0.498	4.57
Hibonite-(Fe)	Fe^2+^Al_12_O_19_	0.436	4.57
Ilmenite	Fe^2+^Ti^4+^O_3_	0.451	4.57
Kamiokite	Fe^2+^_2_Mo^4+^_3_O_8_	0.343	4.57
Actinolite	Ca_2_(Mg_4.5-2.5_Fe^2+^_0.5-2.5_)Si_8_O_22_(OH)_2_	0.437	4.00
Ankerite	CaFe^2+^(CO_3_)_2_	0.486	4.00
Arsenopyrite	Fe^3+^(AsS)^3-^	0.171	4.00
Beaverite-(Cu)	PbCu^2+^Fe^3+^_2_(SO_4_)_2_(OH)_6_	0.247	4.00
Berthierite	Fe^2+^Sb^3+^_2_S^2-^_4_	0.179	4.00
Bornite	Cu^1+^_5_Fe^3+^S^2-^_4_	0.197	4.00
Chalcopyrite	Cu^1+^Fe^3+^S^2-^_2_	0.197	4.00
Chloritoid	Fe^2+^Al_2_OSiO_4_(OH)_2_	0.331	4.00
Cubanite	Cu^1+^Fe^2+^Fe^3+^S^2-^_3_	0.179	4.00
Epidote	Ca_2_Fe^3+^Al_2_(Si_2_O_7_)(SiO_4_)O(OH)	0.407	4.00

Ferro-pargasite	NaCa_2_(Fe^2+^_4_Al)(Si_6_Al_2_)O_22_(OH)_2_	0.458	4.00
Frohbergite	FeTe_2_	0.097	4.00
Grunerite	[box]Fe^2+^_2_Fe^2+^_5_Si_8_O_22_(OH)_2_	0.320	4.00
Hydrobiotite	K(Mg,Fe^2+^)_6_(Si,Al)_8_O_20_(OH)_4_Â·nH_2_O	0.439	4.00
Jarosite	KFe^3+^_3_(S^6+^O_4_)_2_(OH)_6_	0.444	4.00
Lollingite	FeAs_2_	0.123	4.00
Marcasite	FeS_2_	0.241	4.00
Plumbojarosite	PbFe^3+^_6_(SO_4_)_4_(OH)_12_	0.244	4.00
Pyrite	Fe^2+^(S_2_)^2-^	0.241	4.00
Pyrosmalite-(Fe)	Fe^2+^_8_Si_6_O_15_(OH)_10_	0.320	4.00
Schorl	NaFe^2+^_3_Al_6_(Si_6_O_18_)(BO_3_)_3_(OH)_3_OH	0.381	4.00
Siderite	Fe^2+^CO_3_	0.309	4.00
Siderotil	Fe^2+^S^6+^O_4_Â·5H_2_O	0.275	4.00
Stannite	Cu^1+^_2_Fe^2+^Sn^4+^S^2-^_4_	0.167	4.00
Tennantite-(Fe)	Cu^1+^_6_(Cu^1+^_4_Fe^2+^_2_)As^3+^_4_S^2-^_13_	0.160	4.00
Tremolite	[box]Ca_2_Mg_5_Si_8_O_22_(OH)_2_	0.437	4.00
Violarite	Fe^2+^Ni^3+^_2_S^2-^_4_	0.196	4.00
Bonaccordite	Ni^2+^_2_Fe^3+^O_2_(BO_3_)	0.330	3.64
Franklinite	Zn^2+^Fe^3+^_2_O_4_	0.427	3.64
Godlevskite	(Ni,Fe)_9_S_8_	0.196	3.64
Greigite	Fe^2+^Fe^3+^_2_S_4_	0.208	3.64
Jamesonite	Pb^2+^_4_Fe^2+^Sb^3+^_6_S^2-^_14_	0.149	3.64
Lepidocrocite	Fe^3+^O(OH)	0.339	3.64
Natrojarosite	NaFe^3+^_3_(SO_4_)_2_(OH)_6_	0.422	3.64
Nickelskutterudite	(Ni^4+^,Co^3+^,Fe^3+^)As_3_	0.080	3.64
Valleriite	2[(Fe,Cu)S]Â·1.53[(Mg,Al)(OH)_2_]	0.333	3.64
Hisingerite	Fe^3+^_2_Si_2_O_5_(OH)_4_Â·2H_2_O	0.320	3.41
Minnesotaite	Fe^2+^_3_Si_4_O_10_(OH)_2_	0.320	3.41
Neotocite	(Mn^2+^,Fe^2+^)SiO_3_Â·H_2_O	0.338	3.41
Nontronite	Na_0.3_Fe^3+^_2_(Si,Al)_4_O_10_(OH)_2_Â·nH_2_O	0.418	3.41
Allanite-(Ce)	CaCeAl_2_Fe^2+^(Si_2_O_7_)(SiO_4_)O(OH)	0.434	3.37

Segnitite	Pb^2+^Fe^3+^_3_(As^5+^O_4_)(As^5+^O_3_OH)(OH)_6_	0.256	3.35
Isoferroplatinum	Pt_3_Fe	0.155	3.25
Axinite-(Fe)	Ca_4_Fe^2+^_2_Al_4_[B_2_Si_8_O_30_](OH)_2_	0.371	3.20
Corkite	PbFe^3+^_3_(SO_4_)(PO_4_)(OH)_6_	0.227	3.20
Melanterite	Fe^2+^S^6+^O_4_Â·7H_2_O	0.275	3.20
Saponite	(Ca,Na)_0.3_(Mg,Fe^2+^)_3_(Si,Al)_4_O_10_ (OH)_2_Â·4H_2_O	0.453	3.20
Leucophosphite	KFe^3+^_2_(PO_4_)_2_(OH)Â·2H_2_O	0.448	3.11
Strengite	Fe^3+^PO_4_Â·2H_2_O	0.292	3.11
Ulvospinel	Fe^2+^_2_Ti^4+^O_4_	0.451	3.10
Glaucodot	Co^3+^Fe^3+^(AsS)^3-^_2_	0.163	3.07
Columbite-(Fe)	Fe^2+^Nb^5+^_2_O_6_	0.438	3.04
Dufrenite	Ca_0.5_Fe^2+^Fe^3+^_5_(PO_4_)_4_(OH)_6_Â·2H_2_O	0.382	3.04
Tantalite-(Fe)	Fe^2+^Ta_2_O_6_	0.460	3.04
Riebeckite	[box]Na_2_(Fe^2+^_3_Fe^3+^_2_)Si_8_O_22_(OH)_2_	0.403	3.03
Gudmundite	FeSbS	0.179	3.03
Kupcikite	Cu_3.4_Fe_0.6_Bi_5_S_10_	0.164	3.03
Phosphosiderite	Fe^3+^PO_4_Â·2H_2_O	0.292	2.97
Yttrotantalite-(Y)	(Y^3+^,U^6+^,Fe^2+^)(Ta^5+^,Nb^5+^)_2_(O,OH)_4_	0.403	2.95
Stilpnomelane	(K,Ca,Na)(Fe^2+^,Mg,Al,Fe^3+^)_8_(Si,Al)_12_ (O,OH)_36_Â·nH_2_O	0.458	2.93
Almandine	Fe^2+^_3_Al_2_(SiO_4_)_3_	0.382	2.93
Ferro-actinolite	[box]Ca_2_Mg_2.5-0_Fe^2+^_2.5-5_Si_8_O_22_(OH)_2_	0.437	2.92
Vesuvianite	(Ca,Na)_19_(Al,Mg,Fe^3+^)_13_(SiO_4_)_10_ (Si_2_O_7_)_4_(OH,F,O)_10_	0.520	2.92
Ferrimolybdite	Fe^3+^_2_(Mo^6+^O_4_)_3_Â·7H_2_O	0.294	2.91
Annite	KFe^2+^_3_AlSi_3_O_10_(OH)_2_	0.437	2.91
Siderophyllite	KFe^2+^_2_Al(Si_2_Al_2_)O_10_(OH)_2_	0.437	2.91
Staurolite	Fe^2+^_2_Al_9_Si_4_O_23_(OH)	0.331	2.91
Talnakhite	Cu_9_Fe_8_S_16_	0.197	2.91
Tetraferroplatinum	PtFe	0.155	2.91
Tulameenite	CuFePt_2_	0.121	2.91

Argentopentlandite	Ag(Fe,Ni)_8_S_8_	0.169	2.90
Fibroferrite	Fe^3+^S^6+^O_4_(OH)Â·5H_2_O	0.275	2.86
Chamosite	Fe^2+^_5_Al(AlSi_3_)O_10_(OH)_8_	0.363	2.86
Allanite-(La)	CaLaFe^2+^Al_2_(Si_2_O_7_)(SiO_4_)O(OH)	0.437	2.82
Cafarsite	(Ca,Na,[box])_19_Ti^4+^_8_Fe^3+^_4_Fe^2+^_4_(As^3+^O_3_)_28_F	0.523	2.82
Ferberite	Fe^2+^W^6+^O_4_	0.323	2.82
Ferro-tschermakite	[box]Ca_2_(Fe^2+^_3_Al_2_)(Si_6_Al_2_)O_22_(OH)_2_	0.407	2.82
Hemloite	(Ti^4+^,V^3+^,Fe^3+^,Al)_12_As^3+^_2_O_23_(OH)	0.322	2.82
Voltaite	K_2_Fe^2+^_5_Fe^3+^_3_Al(S^6+^O_4_)_12_Â·18H_2_O	0.401	2.82
Gadolinite-(Y)	Fe^2+^Be_2_Y^3+^_2_(SiO_4_)_2_O_2_	0.428	2.82
Berthierine	(Fe^2+^,Fe^3+^,Al)_3_(Si,Al)_2_O_5_(OH)_4_	0.312	2.81
Vermiculite	Mg_0.7_(Mg,Fe^3+^,Al)_6_(Si,Al)_8_O_20_(OH)_4_Â·8H_2_O	0.363	2.81
Foitite	[box](Fe^2+^_2_Al)Al_6_(Si_6_O_18_)(BO_3_)_3_(OH)_3_OH	0.301	2.80
Alluaudite	NaMn^2+^Fe^3+^_2_(PO_4_)_3_	0.470	2.77
Bobfergusonite	[box]Na_2_Mn^2+^_5_Fe^3+^Al(PO_4_)_6_	0.441	2.77
Perloffite	BaMn^2+^_2_Fe^3+^_2_(PO_4_)_3_(OH)_3_	0.421	2.77
Baileychlore	Zn^2+^_5_Al(Si_3_Al)O_10_(OH)_8_	0.349	2.77
Beudantite	Pb^2+^Fe^3+^_3_(As^5+^O_4_)(S^6+^O_4_)(OH)_6_	0.228	2.77
Smythite	(Fe,Ni)_3-3.3_S_4_	0.196	2.75
Iowaite	Mg_6_Fe^3+^_2_(OH)_16_Cl_2_Â·4H_2_O	0.375	2.74
Mountkeithite	(Mg_1-x_Fe^3+^_x_)(S^6+^O_4_)_x/2_(OH)_2_Â·nH_2_O (x < 0.5, n > 3x/2)	0.354	2.74
Pharmacosiderite	KFe^3+^_4_(As^5+^O_4_)_3_(OH)_4_Â·6-7H_2_O	0.448	2.74
Pyroaurite	Mg_6_Fe^3+^_2_CO_3_(OH)_16_Â·4H_2_O	0.354	2.74
Jahnsite-(CaMnFe)	CaMn^2+^Fe^2+^_2_Fe^3+^_2_(P^5+^O_4_)_4_(OH)_2_Â·8H_2_O	0.376	2.74
Scorodite	Fe^3+^As^5+^O_4_Â·2H_2_O	0.293	2.74
Freibergite	Ag^1+^_6_[Cu^1+^_4_Fe^2+^_2_]Sb^3+^_4_S^2-^_12_	0.147	2.74
Jacobsite	Mn^2+^Fe^3+^_2_O_4_	0.449	2.74
Izoklakeite	Pb_26.4_(Cu,Fe)_2_(Sb,Bi)_19.6_S_57_	0.134	2.73
Sternbergite	Ag^1+^Fe_2_S^2-^_3_	0.193	2.73
Rozenite	Fe^2+^S^6+^O_4_Â·4H_2_O	0.275	2.72

Ferro-edenite	NaCa_2_Fe^2+^_5_(Si_7_Al)O_22_(OH)_2_	0.458	2.72
Ferro-hornblende	[box]Ca_2_(Fe^2+^_4_Al)(Si_7_Al)O_22_(OH)_2_	0.407	2.72
Ferronigerite-2N1S	(Al,Fe^3+^,Zn^2+^)_2_(Al,Sn^4+^)_6_O_11_(OH)	0.323	2.72
Hastingsite	NaCa_2_(Fe^2+^_4_Fe^3+^)(Si_6_Al_2_)O_22_(OH)_2_	0.424	2.72
Mawsonite	Cu^1+^_6_Fe^3+^_2_Sn^4+^S^2-^_8_	0.167	2.72
Stannoidite	Cu^1+^_8_Fe^3+^_2_(Fe^2+^,Zn^2+^)Sn^4+^_2_S^2-^_12_	0.164	2.72
Tarkianite	(Cu,Fe)(Re,Mo)_4_S_8_	0.149	2.72
Vinciennite	Cu_10_Fe_4_SnAsS_16_	0.145	2.72
Szomolnokite	Fe^2+^S^6+^O_4_Â·H_2_O	0.275	2.72
Coalingite	Mg_10_Fe^3+^_2_CO_3_(OH)_24_Â·2H_2_O	0.354	2.71
Hydrohonessite	(Ni^2+^_1-x_Fe^3+^_x_)(S^6+^O_4_)_x/2_ (OH)_2_Â·nH_2_O (x < 0.5, n > 3x/2)	0.274	2.71
Oregonite	FeNi_2_As_2_	0.093	2.71
Allanite-(Y)	CaYAl_2_Fe^2+^(Si_2_O_7_)(SiO_4_)O(OH)	0.423	2.70
Ilvaite	CaFe^2+^_2_Fe^3+^OSi_2_O_7_(OH)	0.392	2.70
Tripuhyite	Fe^3+^Sb^5+^O_4_	0.358	2.69
Aegirine-augite	(Ca,Na)(Fe^3+^,Mg,Fe^2+^)Si_2_O_6_	0.485	2.68
Corrensite	(Ca,Na,K)_1-x_(Mg,Fe^2+^,Al)_9_ (Si,Al)_8_O_20_(OH)_10_Â·nH_2_O	0.489	2.68
Ferro-katophorite	NaNaCa[Fe^2+^_4_Al](Si_7_Al)O_22_(OH)_2_	0.458	2.68
Nolanite	V^3+^_8_Fe^3+^_2_O_14_(OH)_2_	0.357	2.68
Vivianite	Fe^2+^_3_(PO_4_)_2_Â·8H_2_O	0.292	2.68
Tapiolite-(Fe)	Fe^2+^Ta_2_O_6_	0.460	2.68
Feruvite	CaFe^2+^_3_(Al_5_Mg)(Si_6_O_18_)(BO_3_)_3_(OH)_3_OH	0.381	2.66
Ferrotitanowodginite	Fe^2+^Ti^4+^Ta^5+^_2_O_8_	0.443	2.65
Ferrowodginite	Fe^2+^Sn^4+^Ta^5+^_2_O_8_	0.394	2.65
Ferrisicklerite	Li_1-x_(Fe^3+^,Mn^2+^)PO_4_	0.460	2.65
Triphylite	LiFe^2+^PO_4_	0.484	2.65
Melkovite	[Ca_2_(H_2_O)_15_Ca(H_2_O)_6_] [Mo^6+^_8_P_2_Fe^3+^_3_O_36_(OH)]	0.368	2.64
Copiapite	Fe^2+^Fe^3+^_4_(S^6+^O_4_)_6_(OH)_2_Â·20H_2_O	0.282	2.64

Coquimbite	Fe^3+^_2_(S^6+^O_4_)_3_Â·9H_2_O	0.275	2.64
Emmonsite	Fe^3+^_2_(Te^4+^O_3_)_3_Â·2H_2_O	0.299	2.64
Qitianlingite	Fe^2+^_2_Nb^5+^_2_W^6+^O_10_	0.355	2.63
Tetraferriphlogopite	KMg_3_(Si_3_Fe^3+^)O_10_(OH)_2_	0.465	2.62
Aegirine	NaFe^3+^Si_2_O_6_	0.514	2.61
Natanite	Fe^2+^Sn^4+^(OH)_6_	0.313	2.61
Mooihoekite	Cu_9_Fe_9_S_16_	0.197	2.61
Lindsleyite	(Ba,Sr)(Zr^4+^,Ca)(Fe^3+^,Mg)_2_ (Ti^4+^,Cr^3+^,Fe^3+^)_18_O_38_	0.503	2.61
Samarskite-(Y)	YFe^3+^Nb^5+^_2_O_8_	0.484	2.61
Yimengite	K(Cr^3+^,Ti^4+^,Fe^3+^,Mg)	0.504	2.61
Halotrichite	Fe^2+^Al_2_(S^6+^O_4_)_4_Â·22H_2_O	0.309	2.60
Rinneite	K_3_NaFe^2+^Cl_6_	0.642	2.60
Tetraferriannite	KFe^2+^_3_(Si_3_Fe^3+^)O_10_(OH)_2_	0.422	2.60
Yukonite	Ca_7_Fe^3+^_15_(As^5+^O_4_)_9_O_16_Â·25H_2_O	0.413	2.59
Potassic-magnesio-arfvedsonite	KNa_2_Mg_4_Fe^3+^Si_8_O_22_(OH)_2_	0.504	2.57
Senaite	Pb^2+^(Mn^2+^,Y^3+^,U)(Fe^2+^,Zn^2+^)_2_ (Ti^4+^,Fe^3+^,Cr^3+^,V^5+^)_18_(O,OH)_38_	0.317	2.57
Ferrosilite	Fe^2+^_2_Si_2_O_6_	0.381	2.55
Ferro-holmquistite	[box]Li_2_(Fe^2+^_3_Al_2_)Si_8_O_22_(OH)_2_	0.410	2.53
Kingsmountite	Ca_3_Mn^2+^Fe^2+^Al_4_(PO_4_)_6_(OH)_4_Â·12H_2_O	0.388	2.53
Kolfanite	Ca_2_Fe^3+^_3_O_2_(As^5+^O_4_)_3_Â·2H_2_O	0.413	2.53
Laueite	Mn^2+^Fe^3+^_2_(PO_4_)_2_(OH)_2_Â·8H_2_O	0.322	2.53
Manganosegelerite	Mn^2+^_2_Fe^3+^(PO_4_)_2_(OH)Â·4H_2_O	0.322	2.53
Mitridatite	Ca_2_Fe^3+^_3_O_2_(PO_4_)_3_Â·3H_2_O	0.412	2.53

Supplementary Table S2: List of Ni-containing minerals with maximum known ages >2.5 Ga that are present in the Fig. 1a and Fig. 1b networks. Weighted Mineral Element Electronegativity Coefficient of Variation (wMEE$_{CV}$). Minerals with maximum known ages (billions of years ago, Ga) greater than 4.33 Ga are from meteorites.

Ni Mineral	RRUFF formula	wMEE$_{CV}$	Maximum Known Age (Ga)
Pentlandite	(Ni,Fe)_9_S_8_	0.165	4.70
Awaruite	Ni_3_Fe	0.021	4.62
Haxonite	(Fe,Ni)_23_C_6_	0.142	4.62
Nickelphosphide	Ni_3_P	0.071	4.62
Reevesite	Ni^2+^_6_Fe^3+^_2_C^4+^O_3_(OH)_16_·4H_2_O	0.246	4.62
Roaldite	(Fe,Ni)_4_N	0.249	4.62
Tetrataenite	FeNi	0.030	4.62
Trevorite	Ni^2+^Fe^3+^_2_O_4_	0.307	4.62
Butianite	Ni_6_SnS_2_	0.142	4.57
Heazlewoodite	Ni_3_S_2_	0.168	4.57
Monipite	MoNiP	0.074	4.57
Nuwaite	Ni_6_GeS_2_	0.141	4.57
Breithauptite	NiSb	0.050	4.00
Gersdorffite-Pa3	Ni^3+^(AsS)^3-^	0.152	4.00
Millerite	NiS	0.211	4.00
Parkerite	Ni_3_(Bi,Pb)_2_S_2_	0.144	4.00

Polydymite	$Ni^{2+}Ni^{3+}_2S^{2-}_4$	0.156	4.00
Shandite	$Pb_2Ni_3S_2$	0.139	4.00
Violarite	$Fe^{2+}Ni^{3+}_2S^{2-}_4$	0.164	4.00
Bonaccordite	$Ni^{2+}_2Fe^{3+}O_2(BO_3)$	0.290	3.64
Bunsenite	$Ni^{2+}O$	0.404	3.64
Gaspeite	$Ni^{2+}CO_3$	0.237	3.64
Godlevskite	$(Ni,Fe)_9S_8$	0.165	3.64
Liebenbergite	$Ni^{2+}_2SiO_4$	0.295	3.64
Maucherite	$Ni_{11}As_8$	0.068	3.64
Nepouite	$Ni^{2+}_3Si_2O_5(OH)_4$	0.267	3.64
Nickeline	NiAs	0.093	3.64
Nickelskutterudite	$(Ni^{4+},Co^{3+},Fe^{3+})As_3$	0.000	3.64
Nimite	$(Ni^{2+},Mg,Al)_6(Si,Al)_4O_{10}(OH)_8$	0.303	3.64
Pecoraite	$Ni^{2+}_3Si_2O_5(OH)_4$	0.267	3.64
Tredouxite	$Ni^{2+}Sb^{5+}_2O_6$	0.243	3.64
Ullmannite	NiSbS	0.162	3.64
Willemseite	$Ni^{2+}_3Si_4O_{10}(OH)_2$	0.267	3.64
Tucekite	$Ni_9Sb_2S_8$	0.149	3.20
Hydrombobomkulite	$(Ni^{2+},Cu^{2+})Al_4(N^{5+}O_3)_2(S^{6+}O_4)(OH)_{12}\cdot 14H_2O$	0.238	3.11
Mbobomkulite	$(Ni^{2+},Cu^{2+})Al_4(N^{5+}O_3,S^{6+}O_4)_2(OH)_{12}\cdot 3H_2O$	0.262	3.11
Melonite	$NiTe_2$	0.054	3.10
Zaratite	$Ni^{2+}_3C^{4+}O_3(OH)_4\cdot 4H_2O$	0.241	3.10
Menshikovite	$Pd_3Ni_2As_3$	0.061	2.91
Argentopentlandite	$Ag(Fe,Ni)_8S_8$	0.165	2.90
Glaukosphaerite	$Cu^{2+}Ni^{2+}CO_3(OH)_2$	0.251	2.90
Nisbite	$NiSb_2$	0.040	2.80
Annabergite	$Ni^{2+}_3(As^{5+}O_4)_2\cdot 8H_2O$	0.239	2.74
Morenosite	$Ni^{2+}S^{6+}O_4\cdot 7H_2O$	0.231	2.74
Arsenohauchecornite	$Ni_{18}Bi_3AsS_{16}$	0.147	2.74

Siegenite	Co^2+^Ni^3+^_2_S^2-^_4_	0.159	2.74
Retgersite	Ni^2+^S^6+^O_4_·6H_2_O	0.231	2.72
Fletcherite	Cu^2+^Ni^3+^_2_S^2-^_4_	0.157	2.72
Vaesite	Ni^2+^(S_2_)^2-^	0.164	2.72
Kambaldaite	NaNi^2+^_4_(C^4+^O_3_)_3_(OH)_3_·3H_2_O	0.263	2.71
Nickelbischofite	Ni^2+^Cl_2_·6H_2_O	0.229	2.71
Nickelblodite	Na_2_Ni^2+^(S^6+^O_4_)_2_·4H_2_O	0.296	2.71
Otwayite	Ni^2+^_2_C^4+^O_3_(OH)_2_·H_2_O	0.246	2.71
Takovite	Ni^2+^_6_Al_2_C^4+^O_3_(OH)_16_·4H_2_O	0.251	2.71
Gillardite	Cu^2+^_3_Ni^2+^Cl^1-^_2_(OH)_6_	0.254	2.71
Nickelhexahydrite	Ni^2+^S^6+^O_4_·6H_2_O	0.231	2.71
Nullaginite	Ni^2+^_2_C^4+^O_3_(OH)_2_	0.250	2.71
Oregonite	FeNi_2_As_2_	0.083	2.71
Ernienickelite	Ni^2+^Mn^4+^_3_O_7_·3H_2_O	0.288	2.68
